# Manganese-Doped Cerium Oxide Nanocomposite Induced Photodynamic Therapy in MCF-7 Cancer Cells and Antibacterial Activity

**DOI:** 10.1155/2019/7156828

**Published:** 2019-09-25

**Authors:** M. Atif, Seemab Iqbal, M. Fakhar-E-Alam, M. Ismail, Qaisar Mansoor, Lubna Mughal, Muhammad Hammad Aziz, Atif Hanif, W. A. Farooq

**Affiliations:** ^1^Department of Physics and Astronomy, College of Science, King Saud University, Riyadh 11451, Saudi Arabia; ^2^Department of Physics, Government College University, Faisalabad 38000, Pakistan; ^3^Institute of Biotechnology and Genetic Engineering, Islamabad, Pakistan; ^4^Department of Physics, Comsats University Lahore Campus, Lahore, Pakistan; ^5^Botany and Microbiology Department, College of Science, King Saud University, Riyadh 11451, Saudi Arabia

## Abstract

In this experimental approach, we explored the structures, morphologies, phototoxicities, and antibacterial activities of undoped and Mn-doped ceria nanocomposite materials, Mn_*x*_Ce_1−*x*_O_2._ The Mn_*x*_Ce_1−*x*_O_2_ nanocomposites were synthesized by employing a soft chemical route. Our prime focus was on the influence of different factors, both physical and chemical, i.e., the concentration of manganese in the product, size of the nanocomposite, drug dose, and incubation time, on the bacterial strains. Different bacterial strains were selected as experimental biological models of the antibacterial activity of the manganese-doped cerium oxide nanocomposite. In addition to the photodynamic response, the adenocarcinoma cell line (MCF-7) was also studied. Based on cell viability losses and bacterial inhibition analyses, the precise mechanisms of apoptosis or necrosis of 5-ALA/PpIX-exposed MCF-7 cells under 630 nm red lights and under dark conditions were elucidated. It was observed that the undoped nanocomposites had lower cytotoxicities and inhibitions compared with those of the doped nanocomposites towards pathogens. The antibacterial activity and effectiveness for photodynamic therapy were enhanced in the presence of the manganese-doped ceria nanocomposite, which could be attributed to the correlation of the maximum reactive oxygen species generation for targeted toxicity and maximum antioxidant property in bacteria growth inhibition. The optimized cell viability dose and doping concentration will be beneficial for treating cancer and bacterial infections in the future.

## 1. Introduction

Metal oxide-based nanomaterials (NMs) are currently popular in multidisciplinary applications. Owing to their biosafe nature, NMs have excellent anticancer and antibiotic properties [1]. Many metal oxide NMs, such as those based on zinc, tin, ferric/ferrous, and cobalt oxides, have been used for therapeutic and diagnostic purposes [1, 2]. Metal oxide nanoparticles with rare earth compounds have potential applications in industry and at commercial levels, e.g., as polishing agents, sunscreen compounds, fuel cell, sensors, and catalysts in automobiles. Their idiosyncratic characteristics have made their mark in biomedicine, nanotherapy, and diagnostic treatments [3–8]. The generation of singlet oxygen is the cause of malignant cell proliferation owing to a higher concentration level of free toxic radicals of oxygen and hydroxyl ions [9].

Several recent studies have demonstrated that cerium oxide NMs have anti-inflammatory, noninvasive, unique electronic configuration, and oxidative stress features. This results in the production of reactive oxygen species (ROS) at the microvascular stage level owing to their natural reduction and oxidation reactions towards the cell line. Their oxidative nature also inhibits the growth of bacteria [10, 11]. In addition, it has been observed that the generation of ROS depends on the level of defects caused by oxygen vacancies in the crystal structure of the nanoparticles. These defects and other physicochemical factors can be introduced and enhanced by different strategies, including annealing via heat, ion beam radiation, and selective metal ion doping. Among these, metal ion doping is an effective and easy tool used to modify the electronic, optical, magnetic, and biomedical features [12].

One study revealed that manganese (Mn) has the ability to increase the chemistry effect, thus elevating the ROS level and Fenton reactions, which form the basis of photodynamic therapy (PDT) [13]. In a similar way, the nitrogen species and scavenging ROS can also be controlled by the presence of the mixed valence state of ceria Ce^3+^/Ce^4+^. This reportedly acts against many bacteria, pathogens, and inflammations owing to this antioxidant property [14]. The Mn dopant can also induce magnetism in the host matrix (CeO_2_) providing a good platform for spintronics, as well as targeted cancer therapy and diagnostics [15]. In contrast, a greater particle size with less doping provides minimal toxicity and the inhibition of bacteria. Therefore, the nature of the doping and quantity play a fundamental role in nanoceria as an antioxidant and oxidant for treating bacteria and cancerous pathogens, respectively. The surface ratio (valence state) of cerium oxide is influenced by physicochemical factors. Therefore, the synthesizing method plays a pivotal role in enhancing the biological and toxicity activity of NPs [16].

**Figure 1:**
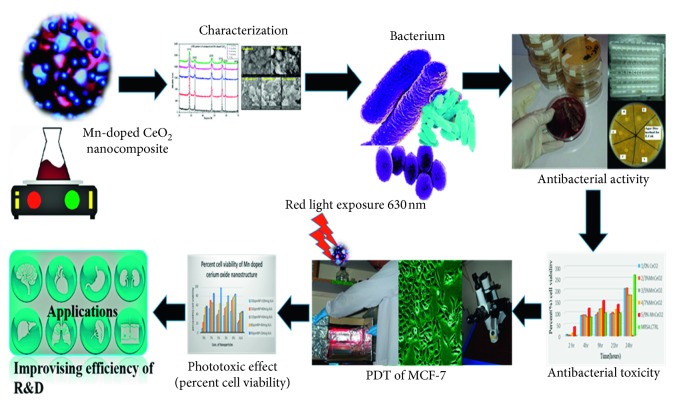
Flowchart of the photodynamic and antibacterial activity of the Mn-doped cerium oxide nanocomposite.

We synthesized Mn^3+^-doped cerium oxide Mn_*x*_Ce_1−*x*_O_2_ nanocomposites with different molar concentrations of Mn as the dopant through the soft chemical pathway of coprecipitation. The material was then characterized using different analytical tools to obtain its structural, morphological, optical, antibacterial, and anticancer features against various bacteria including *Escherichia coli* (*E. coli*), *Staphylococcus aureus* (MRSA), *Pseudomonas aeruginosa* (*P. aeruginosa*), and the adenocarcinoma cell line (MCF-7), as illustrated in Figure 1. In this study, we will explore new strategies to combat breast cancer and the inhibition of growth of various bacterial infections. Mn-doped cerium oxide is an excellent candidate for applications in nanobiology and regenerative nanomedicine in the in vitro cell line and zone of inhibition (ZOI) and is useful for optical density measurements of various bacteria [17].

## 2. Materials and Methods

High-grade chemical salts of Merck and Sigma-Aldrich were used in this experiment.

### 2.1. Chemical Synthesis of Mn-Doped CeO_2_ Nanocomposite

**Figure 2:**
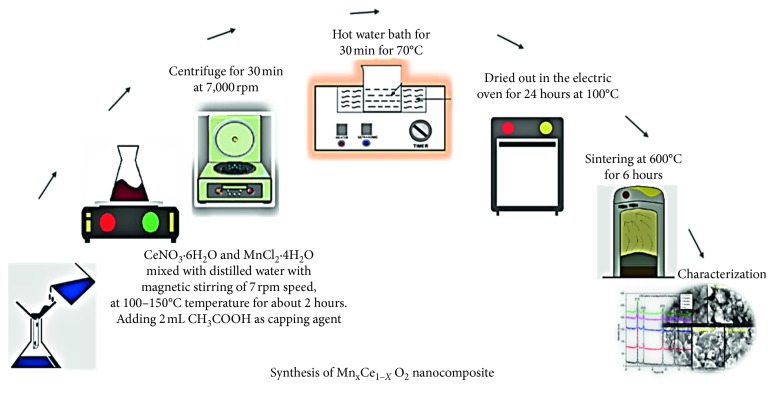
Schematic illustration of the synthesis of the Mn-doped cerium oxide nanocomposite.

The soft chemical route was employed to synthesize Mn_*x*_Ce_1−*x*_O_2_ (where *x* = 0, 3, 5, 7, and 9%) nanocomposites. Cerium nitrate (CeNO_3_·6H_2_O) and manganese chloride (MnCl_2_·4H_2_O) were used as starting materials. Five different molar samples were prepared in distilled water. Each sample solution was placed on a magnetic stirrer while maintaining 700 revolutions per minute (rpm) with the temperature kept in the range of 100–150°C for 2 h to obtain homogeneous solutions. An acetic agent (2 mL of CH_3_COOH) was used as the capping agent and was added dropwise into the parent solution, along with 8 g/100 mL of NaOH base solution (added dropwise) to reach pH 10. The precipitate was then allowed to settle. The initial step for the purification of the solution was to remove the supernatant from the solution using a centrifugation process. The reaction mixtures prepared from the chemicals were kept for 1 day at room temperature for stabilization and subsequently centrifuged at 10000 rpm for 30 min to obtain a clear supernatant and pure nanoparticles. The resulting solution was placed in a water bath for 30 min for further purification. The precipitate was then filtered with filter paper and washed three times with deionized water and methanol/ethanol over the filter paper. The precipitate was dried out in an electric oven for approximately 24 h at 100°C and subsequently ground with a mortar and pestle. The powdered samples were sintered at 600°C for 6 h using an electric furnace [18]. The pellet contained pure NPs. The final product of the calcined materials was analytically studied to obtain different characterizations, as well as the antibacterial and anticancer properties, as shown in Figure 2 [10].

### 2.2. Antibacterial Activity Experimentation

Multidrug-resistant tuberculosis (MDR) bacterium of MRSA, *E. coli*, and *P. aeruginosa* were obtained from clinical isolates of patients affected by urinary tract, throat, and ear infections collected at a microbiology lab of a tertiary care hospital in Islamabad.

### 2.3. Nanocomposite Sensitivity Profiling

The nanocomposite susceptibility patterns of the bacterium were determined by disc diffusion on Mueller–Hinton agar (MHA) plates. Discs containing different concentrations of nanocomposites were placed on the surface of the bacterium-inoculated plates. The ZOI was then measured in millimetres after the plates were incubated for 24 h at 37°C. Colonies of pathogens were cultured in Luria–Bertani (LB) agar. This widely used rich medium is popular because it permits the fast growth of many species. Preparation of the culture media included Bacto Tryptone (3 g), yeast (1.5 g), and NaCl (3 g) (Oxoid, UK). All ingredients were mixed in 200 mL of distilled water with 1 h of continuous stirring, 4 N NaOH was added to maintain a pH of 7.5, before 300 mL of the volume was adjusted followed by autoclave [18].

### 2.4. Bacterial Growth Assessment in Luria–Bertani Agar

The LB culture of the strain was detected for its resistance against the nanocomposite combination. The interactions of nanocomposites against different bacterial strains were performed in two ways: the agar disc diffusion method and by utilising the optical density (OD), as reported previously. For the agar disc diffusion method, the LB broth was striped with *E. coli* bacteria 2-3 times to obtain a homogeneous distribution of inoculums. A serial dilution of 20 mg/mL Mn_*x*_Ce_1−*x*_O_2_ nanocomposite stock was used with 100 *µ*L of strain in 5 mL of LB culture. Then, 1–5 *µ*L of colloidal solutions of test samples was poured on the well plates containing *E. coli* bacteria. The synergism of the beta-lactam antibiotics, in combination with the nanocomposite, was evaluated to observe the growth kinetics of the ESBL strain, before being placed in the incubator for 24 h at 37°C to calculate the zone of inhibition around the well [19]. Secondly, an in vitro antibacterial study was performed by the OD method as reported previously. The strains of *E. coli*, *S. aureus*, and *P. aeruginosa* were cultured in LB media with the test compound and incubated at 37°C [20]. For the OD method, a 100 mL sonicated solution of nanocomposites was added to 100 *µ*L of LB, which was poured into 100–200 *µ*L of inoculum (cultured bacteria in LB). The LB medium, bacteria in the LB medium, and nanocomposites in the LB medium were used as the positive controls for assessing the antibacterial activity. The test sample with inoculum was incubated at 37°C for 24 h, before the bacterium growth was measured at different intervals from 2 to 24 h using a NanoDrop spectrometer (Thermo 2000°C) at 600 nm for the OD. The readings were recorded at 2, 4, 6, 8, 22, and 24 h [21, 22].

### 2.5. Anticancer Experimentation and Cell Culturing

Human breast MCF-7 cells were kept in growth media containing Dulbecco's modified Eagle's medium (DMEM) supplemented with antibiotics and 10% heat-inactivated fetal calf serum (GPPS/FBS), penicillin (100 U/mL), and streptomycin (100 *µ*g/mL), in a 37°C humid atmosphere with 5% CO_2_ in a 75 cm^3^/25 cm^3^ flask. The confluence of MCF-7 cells at a concentration of 10^5^ cells per well in a flask was performed, before planting 96-well plates after the trypsinization process, incubating for 24 h, and proceeded for the test materials. The different concentrations of suspension solutions of un- and Mn-doped CeO_2_ nanocomposites (20 *µ*g/mL (0, 3, 5, 7, and 9 at %)) were added to the cells, along with different doses of aminolevulinic acid (ALA) in *µ*L, and incubated for 24 hr, exposed to red light, and further incubated for 24 hr. The cells used as controls were treated without any nanocomposite [23–29].

### 2.6. Photodynamic Therapy

The cells were cultured with various concentrations of nanocomposites with cells and various concentrations of 5-ALA. For the nontreated negative control group (NTC), the fresh culture medium in the absence of any nanoconjugates was poured into the wells. Red light from a 650 nm wavelength LED array with a power of 50 mW/cm^2^ for 25–30 min was applied to the cells [30].

### 2.7. MTT Staining

The cell viability assay was performed using an MTT assay with the OD of the 650 nm ELISA spectrometer for different concentrations of nanocomposites by adding 10 *µ*L (5 mg/mL) MTT to the seeded cells in 96-well plates and incubating for 4 h at 37°C. DMSO was added to a solubilized solution for 2 h at room temperature [1]. The exposure was short term and drastically decreased the percentage of the cell viability with respect to the concentration of nanocomposites and 5-ALA/PpIX dose in the MCF-7 cell line. The inhibition (or percentage of the cell viability) of the pathogen was calculated from the data by using the following equation:

(1) $$\begin{array}{c} \%\text{cell viability}=\frac{{\text{OD}}_{\text{treated }}-{\text{OD}}_{\text{NPcontrol}}}{{\text{OD}}_{\text{untreated control}}-{\text{OD}}_{\text{blank}}}\times 100. \end{array}$$

#### 2.7.1. Apoptosis Detection Analysis

A fluorescence microscope by Zeiss/Germany and propidium iodide/acridine orange (PI/AO) fluorescent dye were used to detect the apoptosis in the MCF-7 cells induced by the manganese-doped ceria nanocomposite [29]. The MCF-7 cells were incubated for 6, 12, and 24 h with the Mn_*x*_Ce_1−*x*_O_2_ nanocomposites and drugs. The cells were centrifuged for 25 min and their supernatant was thrown. In the next step, the cells were stained with 25 *μ*L solution of the dye and placed on glass slides, before a microscopic study of the slides for detection was performed [30].

#### 2.7.2. Characterization Techniques

The size and surface morphology of the nanocomposites were analysed by a Tescan Vega 3 LMU scanning electron microscope. The crystalline structure of the synthesized nanocomposites was analysed by a D8 Advance X-ray diffractometer (Bruker AXS). The average crystallite size of the nanocomposites was calculated using Scherrer's equation and the [111] plane of the crystal, as follows:

(2) $$\begin{array}{c} D=\frac{0.89\lambda }{ß\;\cos\;\theta }. \end{array}$$

Here, *λ* is the wavelength of the incident radiation and ß is the full width half maximum.

## 3. Results and Discussion

### 3.1. Crystallographic Analysis

**Figure 3:**
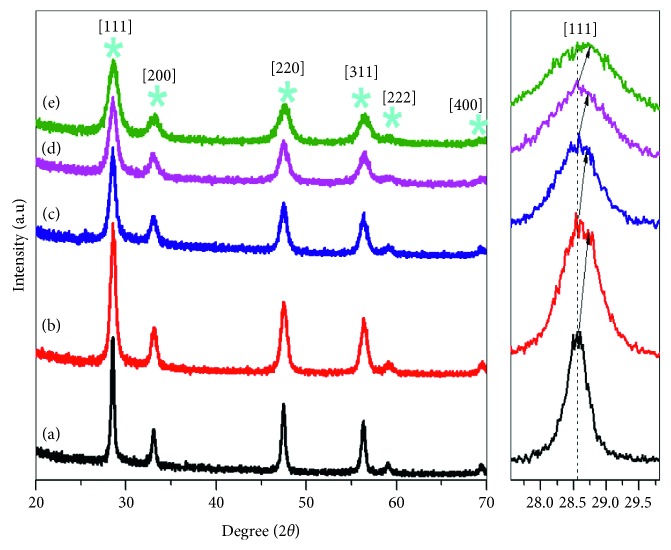
Structural analysis of the Mn_*x*_Ce_1−*x*_O_2_ nanocomposite via the coprecipitation route.

Figure 3 shows the XRD profiles of the synthesized undoped CeO_2_ and doped Mn_*x*_Ce_1−*x*_O_2_ nanocomposites. In the absence of impure peaks, the cubic fluorite single-phase structure according to equation (3) for the undoped CeO_2_ showed the presence of several Bragg peaks at 2*θ* values of 28.58, 32.086, 47.51, 56.49, 59.22, and 69.70° corresponding to [111], [200], [220], [311], [222], and [400], respectively. All planes corresponded to JCPDS card no. 34-0394 [23]. The crystallite size of the undoped CeO_2_ nanocomposite was 8.2 nm (average particle size = 27.2 nm), followed by 11.1 nm for 3% Mn-doped CeO_2_ (average particle size = 27.7 nm), 7.9 nm for 5% Mn-doped CeO_2_ (average particle size = 27.1 nm), 6.7 nm for 7% Mn-doped CeO_2_ (average particle size = 33 nm), and 6 nm for 9% Mn-doped CeO_2_ (average particle size = 45 nm). These were calculated using peak broadening and Debye Scherrer's approximation. The observations were matched with the reported literature [29]. The smallest crystallite size was 27.2 nm for undoped CeO_2_ measured using Scherrer's equation and the [111] plane of the crystal. The substitution of Mn ions into the atomic site of the Ce ion in the matrix of the host CeO_2_ matrix was indicated by the systematic peak [111] shifting towards higher angles with increments in the concentration of Mn content into ceria NPs. Moreover, dopant ions caused lattice contraction as shown by the peak shift. The shift was associated with lattice contraction and distortion due to the replacement of Ce^4+^ ions by smaller ionic radii trivalent Mn^3+^ ions. XRD characterization was employed to investigate the phase purity and average crystallite sizes of the synthesized samples. The enhancement in the specific surface area of the CeO_2_ nanoparticles with Mn may be linked to the crystallite size. It is a well-understood phenomenon that the SSA and crystallite size are inversely related. Hence, the enhancement in the SSA with the increase in the Mn doping concentration may be attributed to the decrease in the crystallite size [1, 22, 28]. The calculated ionic radii of Mn is 0.80 Å, which is less than the calculated ionic radii of Ce (1.01 Å) that caused the decrease in the crystallite size and lattice constant [12, 16]:

(3) $$\begin{array}{c} \frac{1}{d^{2}}=\frac{\sqrt{h^{2}+k^{2}+l^{2}}}{a^{2}}. \end{array}$$

### 3.2. Morphological Analysis

**Figure 4:**
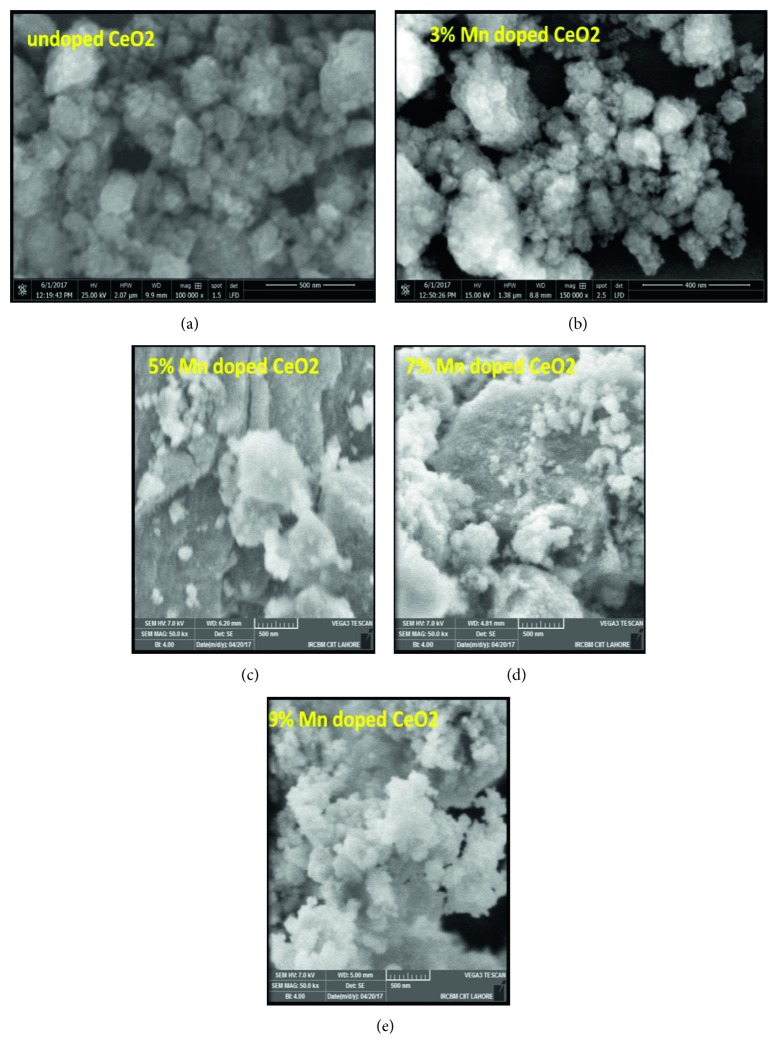
Morphology of the Mn_*x*_Ce_1−*x*_O_2_ nanocomposite: (a) undoped CeO_2_, (b) 3% Mn-doped CeO_2_, (c) 5% Mn-doped CeO_2_, (d) 7% Mn-doped CeO_2_, and (e) 9% Mn-doped CeO_2_.

Figure 4 shows micrographs of the Mn_*x*_Ce_1−*x*_O_2_ samples. The SEM micrographs describe the spherical homogeneous symmetrical nanocomposites of Mn_*x*_Ce_1−*x*_O_2_ with average particle sizes of 27–45 nm [12]. The structure of the synthesized sample changed to heterogeneous (different kind) structures as observed in samples where Mn was doped (7% and 9%) in CeO_2_. Such a structural transformation may be due to the integration of Mn ions into the host CeO_2_ matrix that occurs during chemical reactivity, as well in the lattice establishment of Mn as a dopant in CeO_2_. Thus, the lattice has an elevated proliferation towards a heterogeneous structure [13, 28].

The crystallite size differs from the particle size. A particle may be made up of several different crystallites, an agglomeration of several crystals, or simply one crystallite. Therefore, a particle is not necessarily smaller than a crystallite. XRD, SEM, and TEM are commonly employed to determine if there is a difference between the crystallite and particle sizes. By XRD, Scherrer formulations were used to calculate the crystallite size. SEM was used to calculate the grain/particle size by statistically calculating the particle size using a calibrated SEM image. The average width and length of the images for more than 400 measurements were calculated by the original SEM and ImageJ software.

### 3.3. Antibacterial Analysis (OD + ZOI)

**Figure 5:**
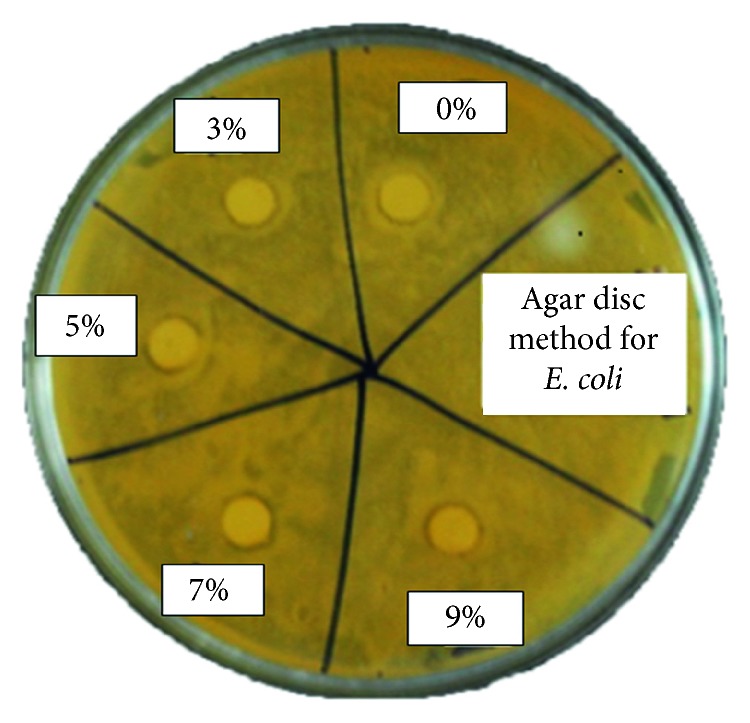
Antibacterial zone of inhibition assay conducted against *E. coli* bacterium to study the efficacy of Mn_*x*_Ce_1−*x*_O_2_ nanocomposites.

**Figure 6:**
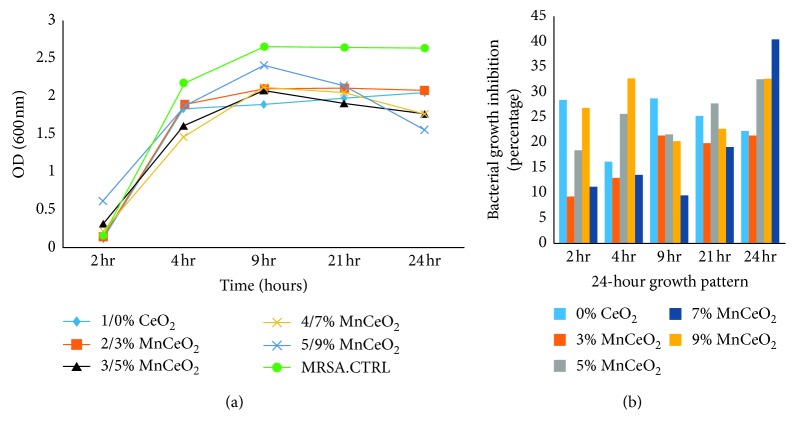
Effect of nanocomposite doping on the growth inhibition of *S. aureus*.

**Figure 7:**
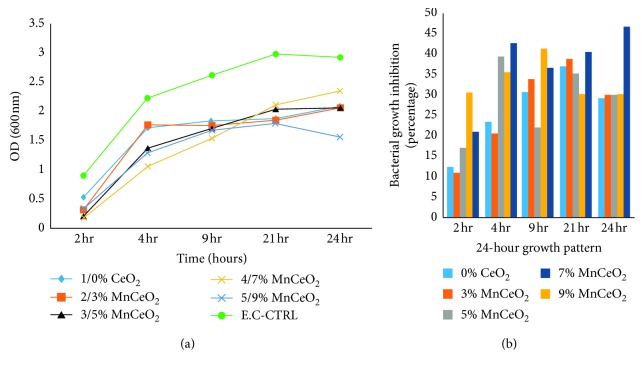
Effect of nanocomposite doping on the growth inhibition of *E. coli*.

**Figure 8:**
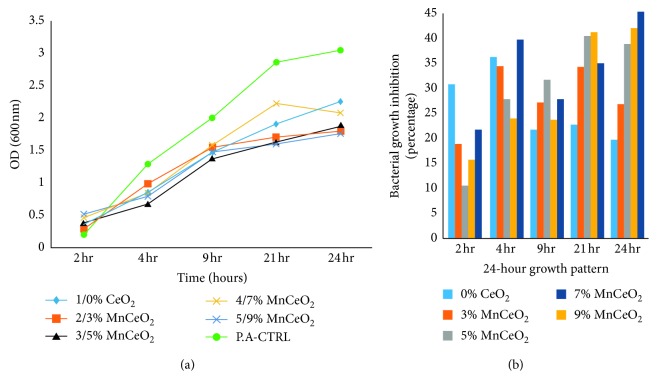
Effect of nanocomposite doping on the growth inhibition of *P. aeruginosa*.

We analysed the antibacterial characteristics of a series of manganese-doped cerium oxide nanocomposites.  Figure 5 shows the antibacterial assay ZOI, conducted for *E. coli* bacterium, when Mn_*x*_Ce_1−*x*_O_2_ nanocomposites were poured into a 400 *μ*L solution of NPs onto each disk. For the *E. coli*, the ZOIs were 8.5, 8, 7, 7, and 2 mm for 9, 7, 5, 3, and 0% of Mn-doped cerium oxide, respectively, as shown in Table 1. Furthermore, two model bacterial strains of Gram-negative bacteria *E. coli* and *P. aeruginosa* and Gram-positive bacteria *S. aureus* were used to study their growth rate using the time-kill assay [13]. The growth inhibition profiles were observed, both with and without the presence of all samples, at different incubation time intervals after 24 h using OD measurements at 600 nm, to obtain the absorbance spectra of the nanocomposites into bacteria [16]. The growth profiles of the bacteria, both with and without nanostructures, are shown in Figures 6–8. The untreated bacteria strains serve as controls for a comparison of results, and it was observed that the prepared nanocomposites inhibited the growth rate of *S. aureus* bacterium. The *S. aureus* (Gram-positive) bacteria was inhibited up to 19, 21, 32, 34, and 40% for 0, 3, 5, 7, and 9% Mn_*x*_Ce_1−*x*_O_2_, respectively, as shown in Figure 6. For the 9% inhibited bacteria growth, a maximum of 40% was reached after 24 hr. For the *E. coli* (Gram-negative), the inhibition rate of the bacteria was 29, 30, 33, 25, and 46% for 0, 3, 5, 7, and 9% Mn_*x*_Ce_1−*x*_O_2_, respectively, against bacterial growth after 24 hr, as shown in Figure 7. For *P. aeruginosa* (Gram-negative), the inhibition rate of bacteria was 19, 26, 38, 41, and 44% for 0, 3, 5, 7, and 9% Mn_*x*_Ce_1−*x*_O_2_, respectively, as shown in Figure 8. The resultant curves show that the inhibition of bacteria increased with increments of manganese doping. A minimum inhibition of bacterial growth was observed for lower Mn content.

An increase in Mn loading into cerium oxide inhibits cell viability [18]. However, differential toxicity occurs because a cell membrane is present along with the cell wall, which results in increased toxicity [19, 21, 31–39]. Many mechanisms have been discussed in relation to the action of nanocomposites towards bacteria [22, 28]. However, it is believed that the small size of the nanocomposites damages the cell wall when in contact, releasing cell/ions constituents by generating reactive oxygen species. Unbalancing of the surface charge is a further mechanism for damaging the bacterial cell wall (negative charge) upon contact with metal oxide nanoparticles/metal ions (positive charge), followed by the internalization of Mn ions into the cell membrane. Therefore, the antibacterial activity of 9% Mn-doped cerium oxide is slightly greater than the other cases, as shown by its ZOI value [25, 26]. These bacteria are hazardous and resistant to antibiotics. Therefore, it is important to produce easy and useful antibacterial agents to control the growth rate of such pathogens.

Gram-negative bacteria (*E. coli* and *P. aeruginosa*) have been found to be relatively more resistant than Gram-positive *S. aureus* bacteria. The bacterial cell walls of *E. coli* and *P. aeruginosa*, for a set of nanoparticles, were tested experimentally. Deciphering the cell wall recycling pathway in *E. coli* is a long and arduous process, compared with that of *P. aeruginosa* due to the difference in the coefficients of permeability [39]. A differential toxicity was observed because a cell membrane was present along with the cell wall. As shown in Figure 7, the incubation rate of the *E. coli* for the 7% Mn-doped sample was very low; however, in the ZOI, it exhibited a promising incubation rate, almost equivalent to 9% Mn-doped ceria. These factors may have affected the 7% activity during the experiment, such as the time lapse between the running of experiments, dissolution in the sample solution, aggregation in the solution, and time of incubation for internalizing the cellular environment. The reduced size of the CeO_2_ nanocomposite doped with Mn (Mn_*x*_Ce_1−*x*_O_2_ at *x* = 1%) contributed to a higher contact surface area [28]. Despite this size, the results of different doping amounts showed different trends, which were most likely associated with a lower photocatalytic activity due to the high turbidity of the cultures resulting in reduced antibacterial activity. Further research is required to address the antimicrobial effects of higher concentrations of metal dopants as the modification of the aspect ratio could reduce the photocatalytic activity, and a higher load of Mn and Ce could increase the cytotoxicity [21, 22].

The OD method was efficient and more accurate than the disc diffusion method. During the ZOI method, the bacterial strips were applied to the surface of the plate at the time of inoculation. The solution of nanoparticles diffused into the medium (disc), resulting in a zone of growth inhibition around the strip. However, there are more associated errors in this procedure compared to the OD method for obtaining accurate results. A UV/Vis spectrophotometer under a wavelength of 600–630 nm was used to conduct the experiments, as well as the NanoDrop spectrometer. The UV/Vis light did not have much of an effect in inhibiting bacteria as observed in the controls, as shown in Figures 6–8 that depict the growth of bacteria without any nanoparticles. Therefore, the activity of such nanocomposites can be enhanced by applying different doses at different incubation intervals and by varying the concentration of dopant into the matrix of the host material [29].

### 3.4. Mechanism of the Interaction of Doped Nanocomposites

**Figure 9:**
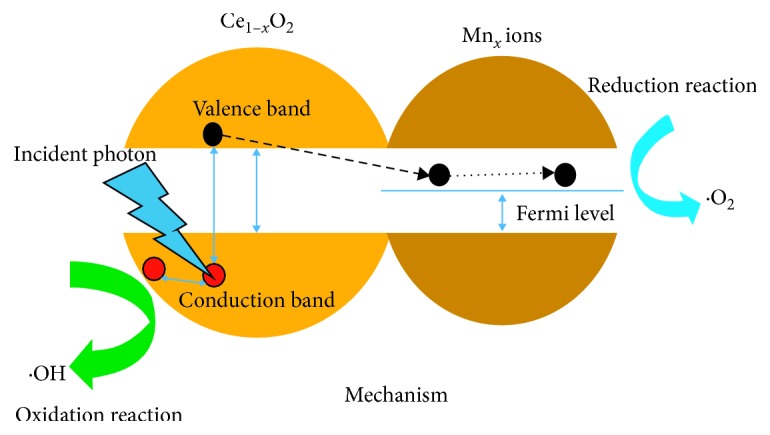
Mechanism of the interaction between doped nanocomposites.

Figure 9 schematically shows the antibacterial activity process mechanism on Mn_*x*_Ce_1−*x*_O_2_ nanocomposites. The coupling or synergetic effects of Mn doping into the host crystal atoms increase the antibacterial activity caused by the generation of reactive oxygen species. Figure 9 shows that electron-hole pairs are generated when UV/Vis light photons are incident on the Mn-doped CeO_2_ nanocomposites, resulting in an excitation process of the electrons from the valence to conduction bands. This produces electron (*e*^−^_cb_) and hole (*h*^+^_vb_) pairs that increase in number within less time. Mn is a good electron acceptor that supplies trapping sites for conduction band electrons, which cause a delay in the recombination process of the electron and hole pairs. Photogenerated electrons transfer to the surface of Mn (dopant) ions from the excited conduction band of CeO_2_, and similarly photogenerated holes transfer to the surface of CeO_2_ NPs. In the meantime, O_2_^∙^ reactive species are produced by the interaction of electrons (*e*^−^_cb_) with dissolved O_2_ on the surface, resulting in an increased production of reactive oxygen species. These migrated holes contribute to the production of ·OH radicals when reacted with chemisorbed H_2_O molecules and form the free radicals ·OH and O_2_^·^, which are the primary cause of cell death and oxidation of organic matter (bacteria and cell walls/membranes) [18, 28]. Moreover, the valence band electrons in these nanocomposites under UV/Vis radiation possess photons of energy greater than or equal to the CeO_2_ bandgap (*e*^−^), which simultaneously excites the conduction band and produces an equal number of holes (*h*^+^) in the valence band. Because the conduction band energy level of CeO_2_ nanoparticles is greater than that of the Fermi level of Mn, electrons can flow from CeO_2_ to Mn. Therefore, oxygen vacancy defects and Mn ions on the surface of CeO_2_ nanoparticles trap electrons and prevent the recombination of *e*^−^–*h*^+^ pairs. The CB electrons then react with dissolved oxygen in the solution to produce superoxide radicals (·O^2−^), while the valence band (VB) holes react with hydroxide ions for the production of hydroxyl radicals (·OH^−^). The free radicals ·O^2−^ and ·OH produced in the reactions can react with organic substances inside bacterial cells to produce bacterial toxins, leading to the death of the bacteria. In particular, the generated H_2_O_2_ easily penetrates the cell membrane and kills the bacteria [11, 12, 17, 20].

The cytotoxicity of metal oxide nanoparticles is associated with several factors, such as the particle size, electrostatic interaction between nanoparticles and cells, and ROS. The nanoparticles have very small sizes, as compared with those of the cells, and can penetrate the cell wall, causing cell damage. Furthermore, it has been reported that CeO_2_ nanoparticles with particle sizes of less than 30 nm remain in cells for a longer time as compared to bigger particles, which leads to harmful effects on the cells [3]. Several types of ROS, such as hydroxyl radicals, singlet oxygen, and H_2_O_2_, could be generated on the surface of nanoparticles by light-induced effects [1]. The enhanced ROS generation could lead to cell death via different mechanisms, such as lipid peroxidation, apoptosis, and cell membrane damage [31–38]. Different types of cells respond differently to CeO_2_ nanoparticles and lead to different levels of ROS generation [15, 28]. The synthesized un- and Mn-doped CeO_2_ nanoparticles may generate different levels of ROS and have different cytotoxicities. It was observed that the ROS generation and cell damage were closely related. Therefore, the differential cytotoxicity of un- and Mn-doped CeO_2_ nanoparticles with up to 7% doping and an overall cytotoxicity of 9% Mn-doped CeO_2_ nanoparticles may have attributed to the different levels of ROS generation.

As for the antibacterial mechanism, several mechanisms of interaction can occur between nanostructures and bacteria cells, such as electrostatic interactions, the attachment of the nanostructures to bacteria cell walls, the release of soluble metal ions, redox reactions, and the generation of ROS. The generation of ROS is highly dependent on the inhibition of the recombination of photogenerated electron-hole pairs. The oxygen vacancies can trap the generated electrons and reduce their likelihood of recombining with holes [17, 20]. It has been reported in the literature that structural defects (oxygen vacancies) in the crystal structure of Mn-doped metal oxide nanostructures lead to a greater level of ROS generation [28]. In our experiment, oxygen vacancies were also found to be enhanced with Mn doping. This argument is still debatable, as similar relations between the oxygen vacancies and level of ROS production for Fe-doped CeO_2_ have been reported; however, no such correlation has been observed for the case of Co-doped CeO_2_ nanoparticles [2, 15]. Hence, more work is required in this regard.

### 3.5. Photodynamic Therapy and Cytotoxic Analysis

**Figure 10:**
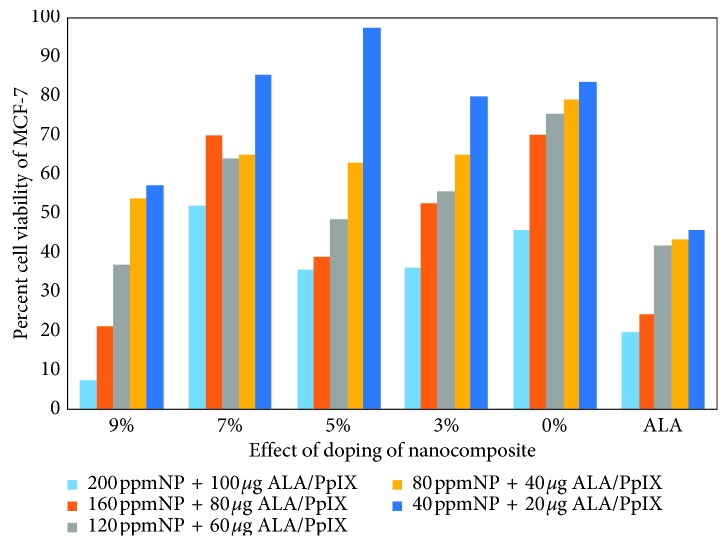
Percent (%) age cell viability at different concentrations of Mn_*x*_Ce_1−*x*_O_2_ nanocomposites into MCF-7 cancer cell line.

The MCF-7 cell line has been used to evaluate the effect of doping and the dose-dependent toxicity of Mn_*x*_Ce_1−*x*_O_2_ nanocomposites [1, 29, 33]. A red light array with a wavelength of 650 nm was used to excite the 5-ALA/PpIX drug to observe the photodynamic effect. The controls were the untreated cells and drug. The viable cells were observed to significantly decrease in number after exposure to red light for 30 min in the presence of the nanocomposite and drug, as compared with the results of the control, treated nanocomposite, and drug-containing cell line. Toxic effects on the cancer cell line were recorded as the drug increased from 40 ppm NP + 20 *µ*g ALA/PpIX to 200 ppm NP + 100 *µ*g 5-ALA/PpIX. Cellular apoptosis was induced by the 9% Mn-doped cerium oxide nanocomposite exposed by 5-ALA/PpIX and NPs as high as 200 ppm NP + 100 *µ*g ALA/PpIX, as shown in Figure 10. Therefore, 9% Mn-doped CeO_2_ nanocomposites are more beneficial for treating MCF-7. The PDT procedure confirmed that the toxicity of the nanocomposites with 5-ALA/PpIX is concentration (doping) and dose dependent because a proper doping concentration and dose provide a high probability of cell necrosis in the presence of light. Moreover, the same concentration of the drug on its own conjugated with the nanocomposite and drug. As shown in Figure 10, for the case of 9% doping and 60 *µ*g 5-ALA/PpIX, a lower amount of the drug could be used for therapy. It was observed that the 9% Mn_*x*_Ce_1−*x*_O_2_ nanocomposite had a 6 nm crystallite size and thus was retained in the cells for a more prolonged time, compared with the larger particle size nanocomposite, resulting in greater damage to the cells [24]. Moreover, the application of light induced the generation of different ROS, such as H_2_O_2_, hydroxyl radicals, and singlet oxygen, upon the nanocomposites entering these cells, causing damage to the cell membrane followed by apoptosis and oxidative degradation of lipids due to the increased ROS production. The reactive oxygen species, electrostatic interaction between cells, nanocomposite, and size and shapes of the particle concentration are all factors related to the cytotoxicity of the metal oxide nanocomposites. Cell damage caused by smaller sized nanoparticles by their internalization through the cell wall and into the cell contents/ions and the selective toxicity of the prepared nanoparticles are due to the differential levels of ROS production caused by different sized particles and doping effects [16, 31, 32]. Hence, the differential cytotoxicity/phototoxicity of the un- and Mn-doped CeO_2_ nanocomposites of up to 7 and 9% doping may correspond to the differential generation of ROS levels.

### 3.6. Determination of IC50 Value

**Figure 11:**
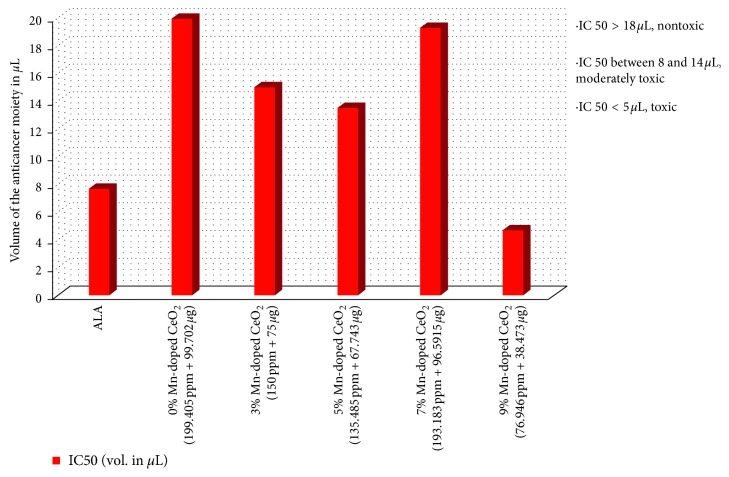
Anticancer moieties: the nanocomposite and drug combined concentrations are described with the independent multiple measurements.

IC50 is the pharmacological measurement of the efficiency of a substance/compound/drug in inhibiting the growth of a targeted biological growth function. It is a quantitative measurement to indicate a specific concentration of drug/nanocomposite required to inhibit a targeted biological process by half (50%), such as cellular or organelle structures. In our research, IC50 was the determination of the killing of 50% cancerous cells (in vitro) caused by the concentration of the drug/nanocomposite and was taken as a cut off for the nanocomposite/drug toxicity against the cell line. A threshold of the IC50 values was obtained by plotting a dose-response curve of the volume of the anticancer moiety in microlitres (percentage of the cell viability) along the *y*-axis and the concentration of the drug/nanocomposite on the *x*-axis. In Figure 11, the IC50 data were analysed by calculating the productivity of the heterogeneous data of the experiment. All concentrations of Mn_*x*_Ce_1−*x*_O_2_ nanocomposites with independently measured IC50 values and age cell viability percentages were extracted, and the liability of the measurements was analysed. The calculations involved the taking of the average of two mean values. The distribution of the IC50 values, shown in Figure 11, was slightly skewed to the right. Moreover, the nanocomposite was categorized as nontoxic, moderately toxic, or toxic if the calculated IC50 was greater than 18 *µ*L, between 8 and 14 *µ*L, or less than 5 *µ*L, respectively.

### 3.7. Apoptosis Detection Analysis

**Figure 12:**
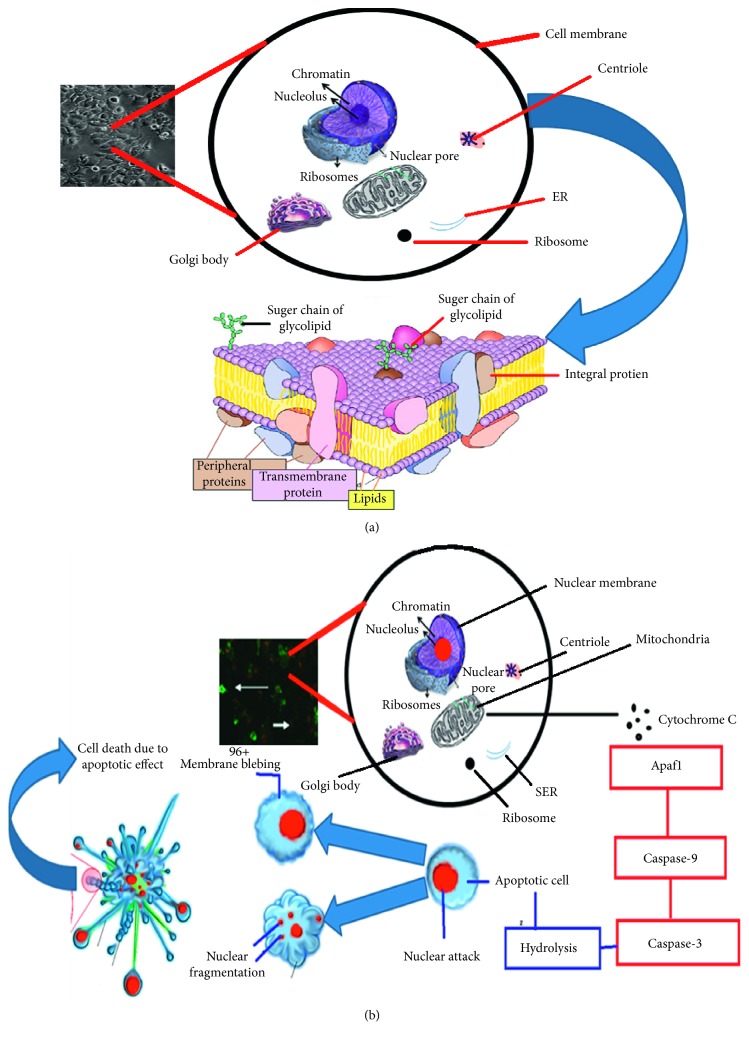
(a) Normal metabolic process of MCF-7 cells with healthy plasma membrane and the control endocytosis process without Mn-doped cerium oxide accumulation. (b) MCF-7 cells death process via the apoptotic effect when treated with Mn-doped cerium oxide (9%). MCF-7 cell death occurred due to programmable cell death via plasma membrane damaging and nuclear fragmentation.

Figure 12 depicts the normal/control metabolic activity and routine endocytosis/exocytosis process schematic for the MCF-7 cells cultured as the control without exposing any of the nanostructures to light irradiation. A schematic of the normal/healthy MCF-7 cells is shown in Figure 12(a). In contrast to the case when the MCF-7 cells were exposed to 9% Mn-doped cerium oxides, sudden cell death occurred via the apoptosis effect (the production of cytochrome, caspase-9, and caspase-3 resulting in the hydrolysis process leading to cell blabbing, plasma membrane rupture, and nuclear fragmentation). The detailed apoptotic mechanism and relevant photochemical reaction are illustrated in Figure 12(b). It was concluded that 9% Mn-doped cerium oxide provided satisfactory favourable effects for cancer treatment. This is in good agreement with previously reported studies [29–39].

## 4. Conclusion

Mn_*x*_Ce_1−*x*_O_2_ nanocomposites were successfully prepared by a facile chemical coprecipitation method. Microstructural studies of Mn_*x*_Ce_1−*x*_O_2_ via XRD and SEM verified their stability in the cubic fluorite phase and nanospherical structure. Mn doped with CeO_2_ exhibited ample antibacterial activities. The 9% doping of Mn in CeO_2_ exhibited a 40% inhibition of the bacterial growth of *S. aureus*, 46% inhibition against *E. coli*, and 44% inhibition against *P. aeruginosa*. Moreover, 9% Mn-doped ceria decreased the viability of MCF-7 cancerous cells by a percentage viability of 7.33. Their toxic effect against cancerous pathogens provides a nanoplatform as a drug delivery agent. It is expected that different structures of nanocomposites and varying amounts of these nanocomposites can increase the effectiveness against bacterial diseases.

## Figures and Tables

**Table 1:** Antibacterial activity of undoped and Mn-doped cerium oxide nanocomposites (zone of inhibitions (mm)).

Bacteria	9%	7%	5%	3%	0%
*E. coli*	8.5	8	7	7	2

## Data Availability

The data used to support the findings of this study are available from the corresponding author upon request.
